# The bearing capacity factor *N*_*γ*_ of strip footings on *c*–*ϕ*–*γ* soil using the method of characteristics

**DOI:** 10.1186/s40064-016-3084-6

**Published:** 2016-09-05

**Authors:** Dongdong Han, Xinyu Xie, Lingwei Zheng, Li Huang

**Affiliations:** 1 Research Center of Coastal and Urban Geotechnical Engineering, Zhejiang University, Hangzhou, 310058 China; 2 School of Civil Engineering and Architecture, Ningbo Institute of Technology, Zhejiang University, Ningbo, 315100 China; 3 MOE Key Laboratory of Soft Soils and Geoenvironmental Engineering, Zhejiang University, Hangzhou, 310058 China

**Keywords:** Bearing capacity, Strip footing, The method of characteristics, Numerical analysis, Shallow foundation

## Abstract

**Background:**

The method of characteristics (also called as the slip-line method) is used to calculate the bearing capacity of strip footings on ponderable soil. The soil is assumed to be a rigid plastic that conforms to the Mohr–Coulomb criterion. The solution procedures proposed in this paper is implemented using a finite difference method and suitable for both smooth and rough footings. By accounting for the influence of the cohesion *c*, the friction angle *ϕ* and the unit weight *γ* of the soil in one failure mechanism, the solution can strictly satisfy the required boundary conditions.

**Results:**

The numerical solution of *N*_*γ*_ are consistent with published complete solutions based on cohesionless soil with no surcharge load. The relationship of *N*_*γ*_ between smooth and rough foundations is discussed which indicates that the value of *N*_*γ*_ for a smooth footing is only half or more of that for a rough footing. The influence of *λ* (*λ* = (*q* + *c*cot *ϕ*)/*γB*) on *N*_*γ*_ is studied. Finally, a curve-fitting formula that simultaneously considers both *ϕ* and *λ* is proposed and is used to produce a series of *N*_*γ*_ versus *λ* curves.

**Conclusions:**

The surcharge ratio *λ* and roughness of the footing base both have significant impacts on *N*_*γ*_. The formula for the bearing capacity on *c*–*ϕ*–*γ* soil can be still expressed by Terzaghi’s equation except that the bearing capacity factor *N*_*γ*_ depends on the surcharge ratio *λ* in addition to the friction angle *ϕ*. Comparisons with the exact solutions obtained from numerical results indicate that the proposed formula is able to provide an accurate approximation with an error of no more than ±2 %.

## Background

The equation for the bearing capacity of a rigid strip footing subjected to a vertical load is commonly expressed as

1 $$q_{u} = cN_{c} + qN_{q} + \frac{1}{2}\gamma BN_{\gamma } ,$$

where *q*_*u*_ is the ultimate bearing capacity; *c*, *q*, *γ* and *B* are respectively the cohesion of the soil, the equivalent surcharge load at the footing base, the unit weight of the soil and the width of the footing; *N*_*c*_, *N*_*q*_ and *N*_*γ*_ represent the bearing capacity factors related to *c*, *q* and *γ*, respectively. Equation (1) was proposed by Terzaghi (1943) and assumes that the factors *N*_*c*_, *N*_*q*_ and *N*_*γ*_ can be obtained by superposition. The soil is treated as weightless when computing *N*_*c*_ and *N*_*q*_ (i.e., *q* ≠ 0, *c* ≠ 0, *γ* = 0) and as ponderable but having no cohesion or surcharge when calculating *N*_*γ*_ (i.e., *q* = 0, *c* = 0, *γ* ≠ 0). The errors caused by this superposition have been discussed by many researchers (Bolton and Lau 1993; Davis and Booker 1971; Griffiths 1982). The superposition approach has been concluded to lead to over-conservative results, which are on the safe side of a design. However, the bearing capacity determined by superposition method superimposes two failure mechanisms, which is different from the real situation. Therefore, it is necessary to calculate the bearing capacity with one failure mechanism to obtain the exact results.

When the bearing capacity is computed on general *c*–*ϕ*–*γ* soil without superposition and the result is still written in the form of Eq. (1), some researchers have found that the value of *N*_*γ*_ relates to not only the soil friction angle *ϕ* but also to other parameters, such as *q*, *c*, *γ* and *B*. Cox (1962) revealed that the parameters associated with stress characteristic equations are *ϕ* and a dimensionless parameter *G*(*G* = *γB*/2*c*) for a smooth footing without surcharge. Chen (1975) introduced a foundation depth and width ratio, *D*/*B*, and computed the changes in *N*_*γ*_ with the *D/B* for different internal friction angles. Xiao et al. (1998) calculated the bearing capacity using the method of characteristics(MOC) and revealed that *q*, *c*, *γ* and *B* all affect *N*_*γ*_, and *N*_*γ*_ is only affected by *ϕ* and *γB*/(*c* + *q*tan *ϕ*) when the load is vertical. Michalowski (1997) and Silvestri (2003) studied the influence of *c*/*γB* and *q*/*γB* on *N*_*γ*_ using the limit analysis method and the limit equilibrium method, respectively. Their research demonstrated that for a given *ϕ*, the value of *N*_*γ*_ significantly changes with *c*/*γB* or *q*/*γB*. Zhu et al. (2003) showed that *N*_*γ*_ is not only related to the friction angle *ϕ* but also to the surcharge ratio *λ* (*λ* = (*q* + *c*cot *ϕ*)/*γB*). Sun et al. (2013) noted that there are two types of failure mechanisms for rough footings, and whether the trapped non-plastic wedge traverses the footing edge depends on the surcharge ratio *λ*. Sun et al. (2013) also studied the variation of *N*_*γ*_ with *ϕ* when *λ* equals the critical surcharge ratio *λ*_c_. The researchers above studied different factors influencing *N*_*γ*_, but none of these studies proposed a formula to calculate *N*_*γ*_.

The MOC is one of main methods applied in the bearing capacity issue which has been discussed by many researchers (Bolton and Lau 1993; Lundgren and Mortensen 1953; Martin 2003; Sokolovskii 1965). The classical bearing capacity factor *N*_*γ*_ when *q* = 0, *c* = 0, *γ* ≠ 0 by MOC is calculated to a high degree of precision and is proven to be exact by checking for coincident lower and upper bounds, and by extending the lower bound stress field throughout the semi-infinite soil domain (Martin 2005; Smith 2005).The bearing capacity on general *c*–*ϕ*–*γ* soil is calculated in one failure mechanism and can be therefore treated as exact solution. In this paper, the MOC is employed to calculate the bearing capacity of strip footings on general *c*–*ϕ*–*γ* soil and is implemented with a self-coded finite difference method program. The computation of the bearing capacity is carried out with one failure mechanism instead of using superposition approximation, which avoids assuming the shape of the slip lines. The present procedures for computing both smooth and rough footings are unified, which satisfies all of the requirements of the boundary conditions and the symmetric conditions of the surface footings. The numerical results of *N*_*γ*_ are compared with other published results, and the sources of errors in the other results are discussed. The bearing capacity factor *N*_*γ*_ on general *c*–*ϕ*–*γ* soil is found to be a function of not only the friction angle *ϕ* but also the surcharge ratio *λ*. Then, the effects of the footing base roughness and the surcharge ratio *λ* on *N*_*γ*_ are investigated. Finally, a curve-fitting formula for *N*_*γ*_ that considers *ϕ* and the surcharge ratio *λ* is proposed based on the numerical results.

## Methods

### Characteristic equations at any point in the limit equilibrium state

**Fig. 1:**
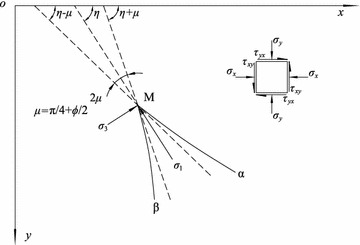
Notation of soil stress at any point in the limit equilibrium state

With the coordinate system depicted in Fig. 1, if a notation is adopted similar to that of Sokolovskii (1965), the normal stresses *σ*_*x*_ and *σ*_*y*_ and the shear stress *τ*_*xy*_ at any point M satisfy the following equilibrium equations:

2 $$\left\{ {\begin{array}{*{20}l} {\frac{{\partial \sigma_{x} }}{\partial x} + \frac{{\partial \tau_{xy} }}{\partial y} = 0} \hfill \\ {\frac{{\partial \sigma_{y} }}{\partial y} + \frac{{\partial \tau_{xy} }}{\partial x} = \gamma } \hfill \\ \end{array} } \right.$$

**Fig. 2:**
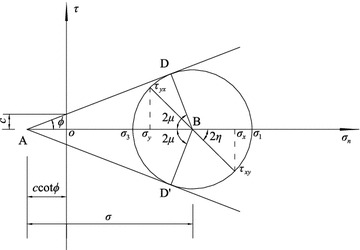
Schematic of the Mohr stress circle in the limit equilibrium state

If the soil satisfies the Mohr–Coulomb criterion, the point M in the limit equilibrium state also satisfies the equation

3 $$\frac{ 1}{ 4}(\sigma_{x} - \sigma_{y} )^{ 2} + \tau_{xy}^{ 2} = \frac{{{ \sin }^{ 2} \phi }}{ 4}(\sigma_{ 1} + \sigma_{ 3} + 2c \cdot { \cot }\,\phi )^{ 2} ,$$

where *σ*_1_ and *σ*_3_ are the major and minor principal stresses, respectively. As shown in Fig. 2, the relations of the principal, normal and shear stresses in the limit equilibrium state can be expressed as the following:

4 $$\left\{ {\begin{array}{*{20}l} {\sigma_{x} = \sigma ( 1+ { \sin }\,\phi \,{\text{cos}}\,2\eta ) - c \cdot { \cot }\,\phi } \hfill \\ {\sigma_{y} = \sigma ( 1- { \sin }\,\phi \cdot {\text{cos}}\,2\eta ) - c \cdot { \cot }\,\phi } \hfill \\ {\tau_{xy} = \sigma \sin \phi \sin 2\eta } \hfill \\ \end{array} } \right.,$$

where *σ* = (*σ*_1_ + *σ*_3_)/2 + *c*cot *ϕ* is defined as a characteristic stress, and *η* is the angle between the major principal stress direction and the horizontal axis *ox*.

According to Eqs. (2) and (4), the differential equations along the α and β characteristic lines can be obtained as follows:

5 $${\text{along}}\;{\text{the}}\;\upalpha\;{\text{characteristic}}\;{\text{line}}\;\left\{ {\begin{array}{*{20}l} {dy = \tan (\eta - \mu )dx} \hfill \\ {d\sigma - 2\sigma \tan \phi d\eta = \gamma (dy - \tan \phi dx)} \hfill \\ \end{array} } \right.$$

6 $${\text{along}}\;{\text{the}}\;\upbeta\;{\text{characteristic}}\;{\text{line}}\;\left\{ {\begin{array}{*{20}l} {dy = \tan (\eta + \mu )dx} \hfill \\ {d\sigma + 2\sigma \tan \phi d\eta = \gamma (dy + \tan \phi dx)} \hfill \\ \end{array} } \right.$$

### Derivation of the finite difference equation

**Fig. 3:**
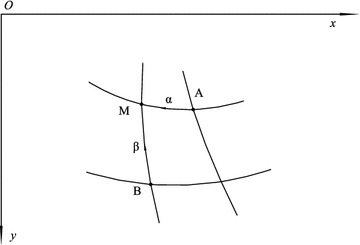
α and β characteristics in the MOC

A theoretical formula for the bearing capacity of strip foundations that considers the weight of the soil is not available. However, a finite difference method is usually used to solve Eqs. (5) and (6). Figure 3 displays the α and β characteristic lines through the point M. If A and B are two points on the α and β characteristic lines near M and the state of these two points is known, the *dx*, *dy*, *dη*, and *dσ* between M and A along the α characteristic line can be approximately expressed as *dx* = *x* − *x*_A_, *dy* = *y* − *y*_A_, *dη* = *η* − *η*_A_ and *dσ* = *σ* − *σ*_A_. By substituting the above formulas into Eq. (5) and taking *η* = *η*_A_ and *σ* = *σ*_A_, the finite difference equations are written as follows:

7 $$\left\{ {\begin{array}{*{20}l} {y - y_{\text{A}} = (x - x_{\text{A}} )\tan (\eta_{\text{A}} - \mu )} \hfill \\ {\sigma - \sigma_{\text{A}} - 2\sigma_{\text{A}} (\eta - \eta_{\text{A}} )\tan \phi = \gamma \left[ {(y - y_{\text{A}} ) - (x - x_{\text{A}} )\tan \phi } \right]} \hfill \\ \end{array} } \right.$$

Likewise, the finite difference equations along the β characteristic line at point M are

8 $$\left\{ \begin{array}{l} y - y_{\text{B}} = (x - x_{\text{B}} )\tan (\eta_{\text{B}} + \mu ) \hfill \\ \sigma - \sigma_{\text{B}} + 2\sigma_{\text{B}} (\eta - \eta_{\text{B}} )\tan \phi = \gamma \left[ {(y - y_{\text{B}} ) + (x - x_{\text{B}} )\tan \phi } \right] \hfill \\ \end{array} \right.$$

The expressions for *x*, *y*, *η* and *σ* at M can be determined from Eqs. (7) and (8).

9 $$x = \frac{{x_{\text{A}} \tan (\eta_{\text{A}} - \mu ) - x_{\text{B}} \tan (\eta_{\text{B}} + \mu ) - (y_{\text{A}} - y_{\text{B}} )}}{{\tan (\eta_{\text{A}} - \mu ) - \tan (\eta_{\text{B}} + \mu )}}$$

10 $$y = \frac{1}{2}(y_{\text{A}} + y_{\text{B}} ) + \frac{1}{2}\left[ {(x - x_{\text{B}} )\tan (\eta_{\text{B}} + \mu ) + (x - x_{\text{A}} )\tan (\eta_{\text{A}} - \mu )} \right]$$

11 $$\eta = \frac{{ - \sigma_{\text{A}} + \sigma_{\text{B}} + 2(\sigma_{\text{A}} \eta_{\text{A}} + \sigma_{\text{B}} \eta_{\text{B}} )\tan \phi + \gamma \left[ {y_{\text{A}} - y_{\text{B}} + (2x - x_{\text{A}} - x_{\text{B}} )\tan \phi } \right]}}{{2(\sigma_{\text{A}} + \sigma_{\text{B}} )\tan \phi }}$$

12 $$\sigma = \frac{1}{2}(\sigma_{\text{A}} + \sigma_{\text{B}} ) + \sigma_{\text{A}} (\eta - \eta_{\text{A}} )\tan \phi - \sigma_{\text{B}} (\eta - \eta_{\text{B}} )\tan \phi + \frac{1}{2}\gamma \left[ {2y - (y_{\text{A}} + y_{\text{B}} ) + (x_{\text{A}} - x_{\text{B}} )\tan \phi } \right]$$

The accuracy of the solutions derived from Eqs. (9) to (12) depends on the spacing of the characteristic lines and the approximation error. In this paper, the soil beneath half of the footing base is divided into more than 1000 elements to attain results with good accuracy. To reduce the error due to approximating *η* = *η*_A_ and *σ* = *σ*_A_ along the α characteristic line and *η* = *η*_B_ and *σ* = *σ*_B_ along the β characteristic line, an iterative algorithm is used. Set *x*^′^ = *x*, *y*^′^ = *y*, *η*^′^ = *η* and *σ*^′^= *σ* after the first calculation, and replace *η* and *σ* with *η* = (*η*^′^ + *η*_A_)/2 and *σ* = (*σ*^′^ + *σ*_A_)/2 in Eq. (5) and *η* = (*η*^′^ + *η*_B_)/2 and *σ* = (*σ*^′^ + *σ*_B_)/2 in Eq. (6). Recalculate the solutions using the updated formulas, and repeat the above procedure until all of the four components converge. Convergence is achieved when

13 $$\left\{ {\begin{array}{*{20}l} {\left| {x - x^{{\prime }} } \right| \le Er \cdot B} \hfill \\ {\left| {y - y^{{\prime }} } \right| \le Er \cdot B} \hfill \\ {\left| {\eta - \eta^{{\prime }} } \right| \le Er} \hfill \\ {\left| {\sigma - \sigma^{{\prime }} } \right| \le Er \cdot \left| \sigma \right|} \hfill \\ \end{array} } \right.,$$

where *Er* is the allowable error in the calculation and is assumed to be 10^−15^.

The state of the field is determined point by point, and the final point exists at the bottom of the footing, for which *y* and *η* are known. As a result, *x* and *σ* at the final point can be directly solved using Eq. (7) without iteration.

### Computation procedure

**Fig. 4:**
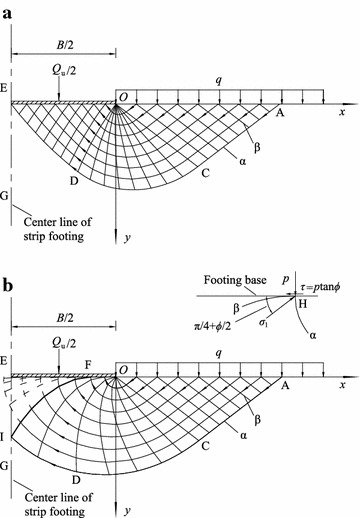
Solution procedure for the MOC. **a** Characteristics of a smooth footing, **b** characteristics of a rough footing

The solution procedure for computing strip footing using the MOC is depicted in Fig. 4. In each figure, OE is the half width of the base, and EG is the center line of the foundation and the axis of symmetry. Note that the edge of footing O is a singularity that can be taken as an α characteristic with zero length. There are a considerable number of *x*, *y*, *η* and *σ* at point O. The number depends on the divisions of the angle of the transition area DOC. The abscissa *x* at any point on the free surface OA is known, and the corresponding *y* and *η* equal 0. According to Eq. (4), *σ* = (*q* + *c*cot)/(1 − sin*ϕ*). Therefore, all of the four components (i.e., *x*, *y*, *η* and *σ*) at the free surface are known. The computation starts from the free surface OA. The solutions for the internal points can be calculated sequentially along the α characteristic. For a smooth footing, the characteristics end at the base. The point at the smooth base has *η* = π/2 and *y* = 0. The calculation is terminated when *x* = −*B*/2.

However, for a rough footing, *η* equals 3π/4 + *ϕ*/2 if the characteristic exists at the footing base. It is impossible for all of the soil under the footing base to be in the yield state because of the roughness. Therefore, the critical problem for rough foundations is determining the boundary of the non-plastic wedge and the yield zone. The α characteristics are assumed to progress to the footing at the beginning, and all the α characteristics thus start from the free boundary and end at the footing base. According to the symmetry requirements, there is no shear stress at the center of the foundation. If the last α characteristic and the centerline of the foundation intersect at point I, the point I will have the properties of *x* = −*B*/2 and *η* = π/2 at the same time. The β characteristic noted as FI in Fig. 4b is the boundary of the non-plastic wedge and the yield zone. The area between FI and the footing is the non-plastic wedge in which the dotted lines in the figure represent nonexistent characteristics. The region FIAO is the yield zone, and the characteristics in this region are real.

From the construction of the characteristic field above, the computational procedure for both smooth and rough footings can be unified. The computation initiates with α characteristics progressing from the free surface to the footing and terminates at the point in which *x* = −*B*/2 and *η* = π/2 simultaneously. The area enclosed by the β characteristic passing through the terminal point and the footing base is the non-plastic zone. The smooth footing is simply the special case presented in Fig. 4b in which the terminal point I coincides with point F and the middle point E of the footing base, which indicates that there is no non-plastic wedge, as shown in Fig. 4a.

The bearing capacity of the smooth and rough footings is given by

14 $$q_{\text{u}} = \frac{{Q_{\text{u}} }}{B} = \frac{{2\int_{\text{OF}} {\sigma_{y} dx} + 2\int_{\text{FI}} {\left( {\sigma_{y} dx - \tau_{xy} dy - dW} \right)} }}{B},$$

where *σ*_*y*_ and *τ*_*xy*_ are the normal stress and shear stress in the *y* direction, respectively, and are obtained from Eq. (4); *dW* is the differential weight of the soil wedge EFI along the curve FI.

For a smooth foundation, the two points I and F coincide at the point E, and Eq. (14) is simplified to

15 $$q_{\text{u}} = \frac{{2\int_{\text{OE}} {\sigma_{y} dx} }}{B}$$

Martin (2003) and Sun et al. (2013) found that there are two types of failure mechanisms for rough footings when constructing the stress field. In one type, no α characteristics progress to the footing base; therefore, a complete non-plastic wedge exists under the footing base. Whereas in the other type, the α characteristics enter the region beneath the footing and result in a partial non-plastic wedge. Sun et al. (2013) stated that the type of failure mechanism depends on *ϕ* and *λ*. The proposed construction method of the characteristics in this paper satisfies all the boundary requirements, and the type of failure mechanism is automatically determined using the computation. Moreover, the computation of the bearing capacity of smooth and rough footings is unified with the same termination condition.

### Equivalent solution of the bearing capacity problem

It is widely accepted that the bearing capacity factors *N*_*c*_ and *N*_*q*_ are correlated with each other through the following formula:

16 $$N_{c} = (N_{q} - 1)\cot \phi$$

When the bearing capacity is computed on general *c*–*ϕ*–*γ* soil without superposition and the result is still written in the form of Eq. (1), the bearing capacity factor *N*_*γ*_ is not the value that computed by superposition method.

Combining Eqs. (16) and (1) gives

17 $$q_{\text{u}} + c\cot \phi = (q + c\cot \phi )N_{q} + \frac{1}{2}\gamma BN_{\gamma }$$

By dividing both sides of Eq. (17) by *γB* and setting *p*_u_ = (*q*_u_ + *c*cot *ϕ*)/*γB* and *λ* = (*q* + *c*cot *ϕ*)/*γB*, the bearing capacity formula is further transformed to

18 $$p_{\text{u}} = \lambda N_{q} + \frac{1}{2}N_{\gamma }$$

Equation (18) is a general solution of the bearing capacity of strip footings that is equivalent to Eq. (1). The *N*_*γ*_ values deduced by exact bearing capacity equal to those by superposition method only when *λ* = 0. To obtain an exact solution of *N*_*γ*_ on general *c*–*ϕ*–*γ* soil, it is necessary to calculate the bearing capacity in the real failure mechanism using a method other than the superposition approximation proposed by Terzaghi. Zhu et al. (2003) computed the bearing capacity factor *N*_*γ*_ of rough strip foundations using the critical slip field method. *p*_u_ and *λ* are defined as the normalized bearing capacity and the surcharge ratio, respectively. The value of *N*_*γ*_ was found to be influenced not only by *ϕ* but also by the surcharge ratio *λ*. However, Zhu et al. (2003) assumed that the inclined angle of the active wedge underneath the footing was π/4 + *ϕ*/2 with respect to the horizontal line, leading to discrepancies between the calculations and the exact solutions. The proposed method in this paper avoids this assumption and results in better numerical results. Moreover, the improved computation extends the application to both a smooth footing and a rough footing. This approach is helpful in attaining a better fitting formula for *N*_*γ*_ based on the exact numerical results.

From Eq. (18), *N*_*γ*_ can be written as

19 $$N_{\gamma } = 2p_{\text{u}} - 2\lambda N_{q}$$

The numerical results of Zhu et al. (2003) implied that *p*_u_ is constant with fixed values for *ϕ* and *λ*. The calculations using the MOC in this paper also confirm this conclusion. Consequently, *N*_*γ*_ is influenced by *ϕ* and *λ* as long as *N*_*q*_ is a function of *ϕ* or *ϕ* and *λ.* The bearing capacity factor *N*_*q*_ is typically regarded as not being influenced by the soil weight with a theoretical formula given by Reissner (1924) as

20 $$N_{q} = {\text{e}}^{{\uppi{ \tan }\phi }} \tan^{2} \left( {\frac{\uppi}{4} + \frac{\phi }{2}} \right)$$

Shield (1954) studied the bearing capacity of strip footings using plastic theory and reached the conclusion that the well-known, closed-form expressions of *N*_*q*_ and *N*_*c*_ given by Reissner (1924) and Prandtl (1921) are exact solutions for weightless soil regardless of the footing roughness. It is most straightforward to use the closed form solutions for *N*_*q*_ and *N*_*c*_ derived for weightless soil and use *N*_*γ*_ to account for all the effects of self weight and its interaction with *q* and *c*, by using the surcharge ratio *λ*.

## Results and discussion

### Comparisons of N_γ_ with other known results

The results of *N*_*γ*_ have been computed by many investigators based on cohesionless soil with no surcharge load (that is, *λ* = 0 in this paper). The present results of *N*_*γ*_ for smooth footings when *λ* = 0 are listed in Table 1. It is noted that the results of *N*_*γ*_ corresponding to *λ* = 0 are computed when *λ* = 10^−10^. The results calculated by other researchers are also presented in Table 1.

**Table 1:** Comparison of the bearing capacity factor *N*_*γ*_ for a smooth footing

*ϕ* (°)	Present method						Bolton and Lau (1993)	Frydman and Burd (1997)	Woodward and Griffiths (1998)	Hjiaj et al. (2005)	Martin (2005)	Smith (2005)	Kumar (2009)
	***λ***												
	0	0.1	1	10	100	10^4^							
5	0.085	0.144	0.210	0.242	0.246	0.248	0.09	–	–	0.0862–0.0914	–	–	0.087
10	0.281	0.438	0.619	0.707	0.721	0.723	0.29	–	0.3	0.283–0.299	0.2809	–	0.282
15	0.699	1.023	1.411	1.605	1.637	1.641	0.71	–	0.7	0.701–0.737	–	0.70	0.699
20	1.579	2.194	2.968	3.375	3.445	3.452	1.60	–	1.5	1.578–1.665	1.579	1.58	1.577
25	3.461	4.607	6.137	6.995	7.145	7.163	3.51	–	3.4	3.454–3.653	–	3.46	3.457
30	7.653	9.816	12.92	14.80	15.16	15.19	7.74	7.9	7.6	7.623–8.078	7.653	7.65	7.644
35	17.58	21.83	28.47	32.88	33.76	33.86	17.8	18.9	–	17.46–18.51	–	17.6	17.55
40	43.19	52.14	67.50	78.96	81.44	81.74	44	42	–	42.77–45.42	43.19	43.2	43.08
45	117.6	138.4	178.1	212.1	220.4	221.3	120	92	–	115.6–123.3	–	118	117.1
50	372.0	427.9	547.6	667.8	701.7	706.2	389	–	–	–	372.0	372	370.0

The present values when *λ* is 0 in Table 1 are equivalent to complete solutions given by Martin (2005) and Smith (2005), which indicates that the results by present method can be treated as exact solutions. The computations provided by Bolton and Lau (1993) or Kumar (2009) using the MOC have little difference compared to present results. Moreover, the results determined by the proposed method are between the upper and lower bounds given by Hjiaj et al. (2005). The *N*_*γ*_ values determined by Woodward and Griffiths (1998) using the finite element method are consistent with those obtained using the MOC. Compared to the calculations given by Hjiaj et al. (2005) and Smith (2005), the results from Frydman and Burd (1997) using fast lagrangian analysis of continua(FLAC) exceed the upper bound when *ϕ* equals 35° and are below the lower bound when *ϕ* equals 40° or 45°, although the errors are small compared with the calculations in this paper.

As inferred in previous section, the value of *N*_*γ*_ depends only on *λ* at a determined *ϕ* if the bearing capacity is calculated without superposition on general *c*–*ϕ*–*γ* soil. The values of *N*_*γ*_ are given in Table 1 as well when *λ* equals to 0.1, 1, 10, 100 and 10^4^. The computations in Table 1 show that the value of *N*_*γ*_ increases with the growth of *λ* if the friction angle is determined. When *λ* equals to 10^4^ or even larger, the value of *N*_*γ*_ is found to approach the theoretical upper bound given by Chen (1975) in the Hill mechanism:

21 $$N_{\gamma } = \frac{1}{4}\tan u\left\{ {\left( {\tan ue^{{1.5\uppi\;f}} - 1} \right) + \frac{3\sin \phi }{{1 + 8\sin^{2} \phi }}\left[ {\left( {\tan u - \frac{\cot \phi }{3}} \right)e^{{1.5\uppi\;f}} + \tan u\frac{\cot \phi }{3} + 1} \right]} \right\},$$

where *u* = π/4 + *ϕ*/2, *f* = tan *ϕ*.

The calculations of *N*_*γ*_ in Table 1 when equals 10^4^ have errors of no more than 0.1 % compared to the solutions by Eq. (21). When the weight of soil decreases to 0, the surcharge ratio *λ* will approach ∞. In this case, the failure surface computed by MOC is consistent with the Hill mechanism. So the upper bound of *N*_*γ*_ in Eq. (21) can be treated as the exact theoretical solution when *λ* = ∞.

Table 2 contains the *N*_*γ*_ results for rough footings. Similar to the results for a smooth footing, *λ* is treated as *λ* = 10^−10^ when *λ* = 0. Some results associated with *λ* = 0 that were published by other researchers are also listed in Table 2.

**Table 2:** Comparison of the bearing capacity factor *N*_*γ*_ for a rough footing

*ϕ* (°)	Present method						Meyerhof (1963)	Bolton and Lau (1993)	Kumbhojkar (1993)	Zhu et al. (2001)	Hjiaj et al. (2005)	Martin (2005)	Smith (2005)	Kumar (2009)
	***λ***													
	0	0.1	1	10	100	10^4^								
5	0.113	0.215	0.376	0.474	0.492	0.495	0.0697	0.62	0.144	0.107	0.115–0.120	–	–	0.114
10	0.433	0.700	1.122	1.387	1.441	1.446	0.367	1.71	0.559	0.453	0.434–0.455	0.4332	–	0.430
15	1.181	1.722	2.579	3.148	3.267	3.282	1.129	3.17	1.520	1.309	1.178–1.234	–	1.18	1.173
20	2.839	3.841	5.458	6.616	6.871	6.904	2.871	5.97	3.641	3.367	2.822–2.961	2.839	2.84	2.822
25	6.491	8.293	11.33	13.70	14.25	14.32	6.765	11.6	8.342	7.864	6.431–6.738	–	6.49	6.458
30	14.75	18.02	23.89	28.94	30.22	30.38	15.67	23.6	19.13	17.58	14.57–15.24	14.75	14.8	14.68
35	34.48	40.61	52.65	64.16	67.32	67.73	37.15	51.0	45.41	40.20	33.95–35.65	–	34.5	34.31
40	85.57	97.93	124.8	153.5	162.3	163.4	93.69	121.0	115.3	97.93	83.33–88.39	85.57	85.6	85.10
45	234.2	262.0	328.7	410.2	438.6	442.7	262.7	324.0	325.3	263.7	224.9–240.9	–	234	232.6
50	742.9	815.2	1008.5	1282.2	1394.5	1412.6	873.9	1052.0	1072.8	824.3	–	742.9	743	736.4

The present values of *N*_*γ*_ when *λ* = 0 are basically treated as exact solutions because they are equal to complete solutions computed by Martin (2005) and Smith (2005). The *N*_*γ*_ results obtained by Bolton and Lau (1993) are much greater than the present values and even exceed the maximum values corresponding to *λ* = 10^4^ when *ϕ* equals 5° or 10°. The large errors are mainly ascribed to the assumption that the trapped wedge beneath the foundation has a base angle of π/4 + *ϕ*/2. Kumar (2009) abandoned this assumption and determined the partly trapped wedge by computation and, consequently, obtained better results. Following Terzaghi’s assumptions, Kumbhojkar (1993) achieved a numerical solution for *N*_*γ*_, and the results are in agreement with Terzaghi’s calculations. Zhu et al. (2001) determined the base angle of the active wedge when *N*_*γ*_ is a minimum using the method of triangular slices, and the corresponding *N*_*γ*_ results are better than those determined by Kumbhojkar (1993). Hjiaj et al. (2005) meshed fine finite elements to determine the yield zones instead using an arbitrary assumption. The errors do not exceed 3.42 % between the rigorous lower and upper bound solutions, and the results are in good agreement with the present calculations.

When *λ* equals to 0.1, 1, 10, 100 and 10^4^, the values of *N*_*γ*_ are also given in Table 2. Similar to smooth footings, the value of *N*_*γ*_ approaches the upper bound in the Prandtl mechanism when *λ* equals to 10^4^. The exact theoretical solution of the upper bound is also given by Chen (1975), which is twice of the value calculated with Eq. (21). The values of *N*_*γ*_ in Table 2 when *λ* equals 10^4^ are basically equal to the theoretical solutions with the errors less than 0.1 %.

### Ratio of N_γ_ for smooth and rough footings

**Fig. 5:**
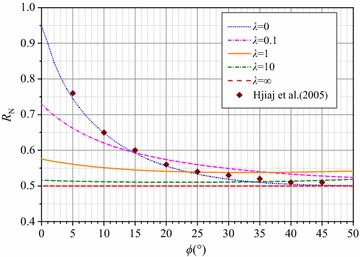
Numerical results of the *R*_N_ ratio versus *ϕ*

The numerical results in Tables 1 and 2 indicate that the *N*_*γ*_ values have large differences for smooth and rough foundations for given *ϕ* and *λ* values. This implies that the roughness of the footing base has a large impact on *N*_*γ*_. Accordingly, the ratio of the *N*_*γ*_ values for smooth and the rough foundations is defined as *R*_N_, i.e.,

22 $$R_{\text{N}} = \frac{{N_{\gamma }^{\text{s}} }}{{N_{\gamma }^{\text{r}} }},$$

where $N_{\gamma }^{\text{s}}$ and $N_{\gamma }^{\text{r}}$ are the bearing capacity factors *N*_*γ*_ for the smooth and rough foundations, respectively, for given *ϕ* and *λ* values. The curves of *R*_N_ versus *ϕ* with different *λ* are plotted in Fig. 5, and the results given by Hjiaj et al. (2005) are also marked in the figure. The computations by Hjiaj et al. (2005) in the case of *q* = 0 and *c* = 0 are in good agreement with the curve for *λ* = 0.

The numerical calculations of *N*_*γ*_ for smooth footings and rough footings reveal that the *N*_*γ*_ value for a smooth footing is only half or more than half of that for a rough footing. Figure 5 also demonstrates that *R*_N_ becomes less sensitive to *ϕ* as *λ* increases. Equivalent to the solution for a granular soil with zero surcharge, the numerical result of *N*_*γ*_ when *λ* equals 0 is a minimum solution with a determined *ϕ*. For a rough foundation, the collapsed surface when *λ* = ∞ is the same as that in the Prandtl mechanism, and the computational result of *N*_*γ*_ equals the closed-form solution deduced in the Prandtl mechanism. Similarly, the *N*_*γ*_ for a smooth footing is identical to the theoretical expression in Hill’s failure mechanism. As stated by Chen (1975), the *N*_*γ*_ in the Prandtl mechanism is exactly twice the value in the Hill mechanism, i.e., *R*_*N*_ = 0.5. The relationship of *R*_N_ and *ϕ* when *λ* = ∞ in Fig. 5 verifies Chen’s judgment.

### Influence of the surcharge ratio on N_γ_

The computations of *N*_*γ*_ also exhibit large discrepancies when *λ* = 0 and *λ* = ∞ at the same *ϕ* regardless of having a smooth or rough footing base. To distinguish the *N*_*γ*_ for different values of *λ*, *N*_*γ*_ is noted as *N*_*γ*,min_ when *λ* = 0 and as *N*_*γ*,max_ when *λ* = ∞. It can be easily inferred that the *N*_*γ*,max_ of a smooth foundation is exactly calculated by Eq. (21) and the *N*_*γ*,max_ of a rough footing is twice of that of a smooth footing. If *K*_N_ is defined as the ratio of *N*_*γ*_ at *λ* = 0 and *λ* = ∞, then

23 $$K_{\text{N}} = \frac{{N_{\gamma ,{\rm min} }^{{}} }}{{N_{\gamma ,{\rm max} }^{{}} }}$$

**Fig. 6:**
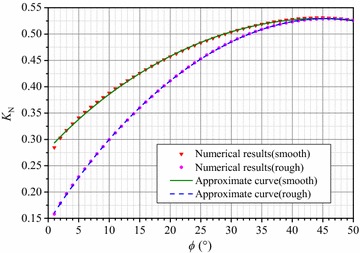
Numerical results and fitting curves of the ratio *K*_N_ versus *ϕ*

Figure 6 reveals the relationship of *K*_N_ to *ϕ* for smooth and rough footings. A smaller *ϕ* results in a larger difference in *K*_N_ between smooth and rough footings. The values of *K*_N_ for smooth and rough foundations are very close when *ϕ* is greater than 40°. Furthermore, the numerical results of *K*_N_ can be approximated using a polynomial expression in the form below

24 $$K_{\text{N}} = \sum\limits_{i = 0}^{n} {a_{i} \tan^{i} } \phi$$

Taking *n* as 4 in Eq. (24), the error between the approximated and numerical values appears to be no more than ±1 % when *ϕ* is greater than 2°. Therefore, the suggested expression for smooth footings can be written as follows:

25 $$K_{\text{N}} = - 0.0654\tan^{4} \phi + 0.345\tan^{3} \phi - 0.747\tan^{2} \phi + 0.715\tan \phi + 0.281$$

Furthermore, the fitting formula for *K*_N_ for a rough footing is given as follows:

26 $$K_{\text{N}} = - 0.0719\tan^{4} \phi + 0.441\tan^{3} \phi - 1.047\tan^{2} \phi + 1.065\tan \phi + 0.142$$

The curves of *K*_N_ versus *ϕ* according to Eqs. (25) and (26) are plotted in Fig. 6, and the curves are in good agreement with the numerical computations.

### Proposed formula of N_γ_

**Fig. 7:**
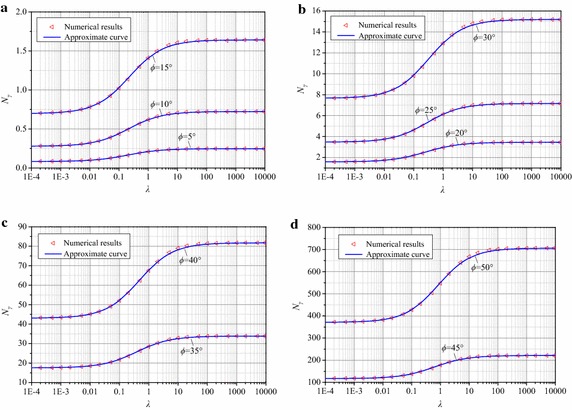
Comparison of *N*_*γ*_ between numerical and approximate results for a smooth footing. **a** *ϕ* = 5°,10° and 15°, **b** *ϕ* = 20°,25° and 30°, **c** *ϕ* = 35° and 40°, **d** *ϕ* = 45° and 50°

**Fig. 8:**
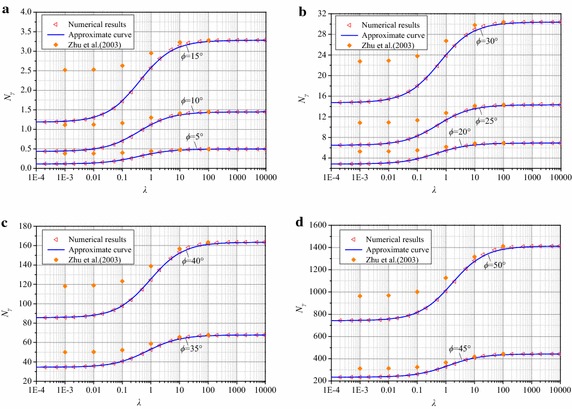
Comparison of *N*_*γ*_ between numerical and approximate results for a rough footing. **a** *ϕ* = 5°,10° and 15°, **b** *ϕ* = 20°,25° and 30°, **c** *ϕ* = 35° and 40°, **d** *ϕ* = 45° and 50°

The bearing capacity factor *N*_*γ*_ is influenced by both *λ* and *ϕ* regardless of Eq. (19) or the numerical calculations using the MOC. The calculations of *N*_*γ*_ related to *λ* with a series of *ϕ* are plotted in Figs. 7 and 8 for smooth footings and rough footings, respectively. The tendency in both figures implies that *N*_*γ*_ tends to gradually decrease to the value *N*_*γ*,min_ when *λ* approaches 0 and to increase to *N*_*γ*,max_ when *λ* is sufficiently large. Based on the numerical results, a general fitting formula for both smooth and rough footings is proposed as

27 $$N_{\gamma } = N_{\gamma ,{\rm min} } + \frac{{N_{\gamma ,{\rm max} } - N_{\gamma ,{\rm min} } }}{{1 + \left( {\frac{{A_{0} }}{\lambda }} \right)^{p} }},$$

where *p* and *A*_0_ are fitting parameters.

As mentioned previously, the *N*_*γ*,max_ of a foundation can be exactly calculated by theoretical solutions. However, the exact closed form solution of *N*_*γ*,min_ is not available when *λ* = 0, although plenty of empirical formulas are given by different researchers. The *N*_*γ*,min_ value is proposed to be calculated by the solution of *N*_*γ*,max_ times *K*_N_ based on Eqs. (25) and (26).

The parameter *p* ranges from 0.75 to 0.8 for both smooth and rough footings. The value of *p* was selected to be 0.75 because the variation of *p* from 0.75 to 0.8 has little effect on *N*_*γ*_. The factor *A*_0_ is a fitting coefficient that is related to *ϕ* and is defined as

28 $$A_{0} = \sum\limits_{i = 0}^{n} {a_{i} \tan^{i} } \phi$$

The value of *A*_0_ has satisfactory accuracy when *n* is 3. The proposed formulas for *A*_0_ are given below:

29 $${\text{for}}\;{\text{smooth}}\;{\text{footings}}\;A_{0} = 0.222\tan^{3} \phi + 0.101\tan^{2} \phi + 0.102\tan \phi + 0.188$$

30 $${\text{for}}\;{\text{rough}}\;{\text{footings}}\;A_{0} = 0.22\tan^{3} \phi + 0.684\tan^{2} \phi - 0.042\tan \phi + 0.354$$

The curves of *N*_*γ*_ versus *λ* for smooth and rough footings are plotted in Figs. 7 and 8, respectively, based on the fitting formula (27). For both smooth and rough footings, the approximate results agree well with the numerical results within errors of ±2 %. Therefore, Eq. (27) is able to estimate *N*_*γ*_ with adequate accuracy.

The *N*_*γ*_ data computed by Zhu et al. (2003) are given in Fig. 8 as well and are much greater than the results of the proposed method when *λ* is less than 10. As mentioned above, the discrepancies in the results are mainly attributed to the assumption that the base angle of the active wedge underneath the footing base equals 45° + *ϕ*/2. The error resulting from that assumption rapidly decreases with increasing *λ*. As seen in Fig. 8, there is little difference between the present values and the results provided by Zhu et al. (2003) when *λ* is greater than 10. Moreover, the theoretical solution of *N*_*γ*_ given by Zhu et al. (2003) is the same as the present value in the case of *λ* = ∞. Similar to the pattern of the present results, the calculations by Zhu et al. (2003) also have an “S” shape for a fixed *ϕ*, which implies that the results can be estimated by expression (27) as well, except *N*_*γ*,min_, *A*_0_ and *p* differ from the values in this paper.

It should be noted the proposed approximate formula of *N*_*γ*_ in Eq. (27) is limited to the classic issue on the bearing capacity of strip footings that the soil is treated as a rigid plastic and obeys Mohr–Coulomb criterion. If the soil beneath the strip footing does not flow Mohr–Coulomb criterion, the proposed method may be no longer applicable. Because the suggested approximate formula is based on the conclusion that the bearing capacity factor *N*_*γ*_ depends on the surcharge ratio *λ* in addition to the friction angle *ϕ*. This conclusion is only valid for Mohr–Coulomb soil. Further research is required whether the conclusion is suitable when the soil meets other yield criteria other than Mohr–Coulomb criterion. A comprehensive research is also needed whether the conclusions and proposed formula in this paper can be extended to circular or rectangular footings.

## Conclusions

The MOC is employed to calculate the exact bearing capacity of strip footings. By considering the influence of *c*, *ϕ* and *γ* with one failure mechanism, the computational procedures for smooth and rough foundations are unified without assuming the failure mechanism. The computations were implemented using a self-coded finite difference program. If the bearing capacity of the footings is calculated using the formula proposed by Terzaghi (1943) and *N*_*q*_ and *N*_*c*_ are obtained using the theoretical solutions given by Prandtl (1921) and Reissner (1924), the value of *N*_*γ*_ is influenced by not only the friction angle *ϕ* but also by the surcharge ratio *λ*. The computations were compared with other published results. The comparisons and analysis indicate the following conclusions:

1. In the case of no overload, the computed *N*_*γ*_ values in this paper for a granular soil are treated as exact solutions because the values are consistent with complete solutions given by Martin (2005) and Smith (2005). Some researchers assume failure surfaces or mechanisms that are not the same as the real state; therefore, their results have considerable errors compared with the exact solutions.
2. The roughness of the footing base has a significant impact on *N*_*γ*_. The ratio of the bearing capacity factor *N*_*γ*_ for smooth foundations and rough foundations, which is *R*_N_, indicates that the value of *N*_*γ*_ for a smooth footing is only half or more of that for a rough footing. The curve of *R*_N_ versus *ϕ* with *λ* = 0 has good agreement with the results given by Hjiaj et al. (2005). A value of *R*_*N*_ equal to 0.5 when *λ* = ∞ supports Chen’s statement that the *N*_*γ*_ in the Prandtl mechanism is exactly twice the value in the Hill mechanism.
3. The surcharge ratio *λ* also significantly affects *N*_*γ*_, and the ratio *K*_*N*_ defined by *N*_*γ*,min_/*N*_*γ*,max_ can be approximately evaluated using a polynomial expression when *λ* = 0 and *λ* = ∞. When *λ* is sufficiently large, the solution of *N*_*γ*_ is demonstrated to approach the upper bound that deduced by Chen (1975) in a closed-form solution. Therefore, *N*_*γ*,max_ is obtained by exact theoretical formula, and thus, *N*_*γ*,min_ can be accurately estimated using *K*_*N*_ and *N*_*γ*,max_.
4. The present *N*_*γ*_ value in the case of *λ* = ∞ is exactly the same as the theoretical solution of *N*_*γ*_ given by Zhu et al. (2003). However, the calculations of Zhu et al. (2003) have obvious errors compared with the present results when *λ* is less than 10 primarily due to the assumption that the base angle of the active wedge underneath the footing base equals 45^°^ + *ϕ*/2. The values of *N*_*γ*_ can be calculated by the approximate formula (27) containing two factors: *ϕ* and *λ*. The discrepancies between the approximate results and the numerical solutions are less than ±2 % for both smooth and rough foundations. Formula (27) is demonstrated to be suitable for evaluating *N*_*γ*_ when considering the factor *λ*.
